# Differential Impacts of HIV status on short-term fertility desires among couples in Rakai, Uganda

**DOI:** 10.1371/journal.pone.0210935

**Published:** 2019-01-24

**Authors:** Xiaoyu Song, Stephanie A. Grilo, Sanyukta Mathur, Tom Lutalo, Robert Ssekubugu, Fred Nalugoda, John S. Santelli

**Affiliations:** 1 Department of Population Health Science and Policy, Icahn School of Medicine at Mount Sinai, New York, NY, United States of America; 2 The Tisch Cancer Institute, Icahn School of Medicine at Mount Sinai, New York, NY, United States of America; 3 Columbia University Mailman School of Public Health, Heilbrunn Department of Population and Family Health, New York, NY, United States of America; 4 Columbia University Mailman School of Public health, Department of Sociomedical Sciences, New York, NY, United States of America; 5 Population Council, Washington D.C., United States of America; 6 Rakai Health Sciences Program (RHSP), Rakai District, Uganda; USC Keck School of Medicine, Institute for Global Health, UNITED STATES

## Abstract

**Background:**

Fertility desires of female and male partners in current relationships are often correlated. We examined the influence of HIV seropositive status of female and male partners on short-term fertility desires in Rakai, Uganda, a setting with high fertility and HIV infection rates.

**Methods:**

Participants were couples (15–49 years old) enrolled in the Rakai Community Cohort Study, from 2011 to 2013 (n = 2,291). Cohen’s kappa coefficient was used to measure the correlation of female and male partners’ short-term fertility desires (measured as ‘wanting a child in the next 12 months’), in both total sample and stratified serostatus groups. HIV serostatus and additional characteristics of female and male partners were included in Poisson regression models to estimate the rate ratios (RR) for each partner’s short-term fertility desires. Individual and partner characteristics included HIV status, partner HIV status, age in years, partner age in years, educational attainment, number of living children, community of residence, and socioeconomic status (SES).

**Results:**

Short-term fertility desires among female and male partners were moderately associated (Kappa = 0.37, p-value<0.001). The association was weakest among female sero-positive and male sero-negative couples (Kappa = 0.29, p-value<0.001). When adjusting for parity and other covariates in the model, women’s short-term fertility desires were significantly associated with their positive sero-status regardless of male partners’ sero-status (adjRR = 1.58, p<0.001 for F+M-; adjRR = 1.33, p = 0.001 for F+M+; in comparison with F-M-). Men’s short-term fertility desires were significantly associated with their positive sero-status, in addition to their female partners’ positive sero-status (adjRR = 1.23 with p-value = 0.022 for F-M+; adjRR = 1.42 with p-value<0.001 for F+M-; adjRR = 1.26 with p-value<0.001 for F+M+; in comparison with F-M-). When the differential effect of parity was included in the model, similar associations remained for both female and male partners when the number of living children was small, but largely reduced when the number of living children was large (3 or more).

**Conclusion:**

Female and male partners in couple dyads demonstrated moderate agreements about short-term fertility desires. The HIV seropositive status of female partners was most strongly associated with short-term fertility desires of both genders, and this association was even stronger for women who had few or no living children.

## Introduction

Reproduction is dyadic in nature. People incorporate their partner’s reproductive desires into their own intentions and adjust their pregnancy-seeking/avoiding behaviors.[1] In Sub-Saharan Africa (SSA), patriarchal gender norms often lead men to be the dominant influencers in decision-making issues related to fertility and family planning,[2] despite women’s central socio-biological roles in the reproduction and procreation processes.[3] Moreover, when partner desires differ, men’s preferences around pregnancy intentions, planning and prevention tend to be more influential than women’s preferences and desires.[4] Studies that fail to address the differentiating roles of men and women in fertility decisions miss opportunities to understand key relationship dynamics related to family planning and reproductive health, potentially leading to inefficient programing aimed at reducing unintended fertility and maximizing women’s reproductive choices.

In contexts of high HIV prevalence and high fertility rates like SSA, fertility-related research has primarily focused on the female perspective, primarily due to the difficulty in collecting partner and couple-level data. These prior studies provide mixed findings about the relationship between fertility desires and HIV status.[5–13] For instance, some studies found that HIV status did not have a significant impact on the desire to have children, while others showed that knowledge of one’s HIV status and use of HIV therapies decreased the desire for pregnancy.[4–12] However, little is known about how women and men’s fertility desires might interplay, differ and/or be similar when factoring in both female and male partners’ HIV status.

A few recent studies have started to incorporate male partner or couple sero-status data in their analysis, but none included population-level data. One study done in South Africa, Tanzania and Ukraine focused on sero-discordant couples, and found that they experienced high levels of discrimination and stigma and often hid their discordant status from family as well as providers–inhibiting the ability to provide HIV prevention.[14] Another study in Uganda included HIV positive women, and demonstrated that there was an association between their partner’s fertility desire and contraceptive use, again underscoring the importance of including men in reproductive health interventions. A recent paper written about couples’ HIV counseling in Rakai, Uganda measured total fertility desire and found sero-positive status to be negatively associated with the couples’ total fertility desires.[15] Moreover, the authors failed to include partner sero-status as a potential influence on the individual’s total fertility desire, in addition to considering other fertility desire variables such as short-term fertility.

In this paper, we examine the influence of HIV seropositive status of both female and male partners in on-going relationships on couples’ short-term fertility desires. The study was conducted in a context of high HIV prevalence and high fertility rates: specifically, Uganda has national adult (ages 15–49) HIV prevalence of 6.5%,[16] and a total fertility rate of 5.7 children per woman.[17] Relative to national levels, the Rakai District in Uganda has an even higher HIV prevalence of 12.2%.[18] Specifically, we aim to assess whether HIV-affected couples (discordant or concordant) are more (or less) likely to want another child within the next 12 months in comparison to HIV-negative couples. We use data from the Rakai Community Cohort Study (RCCS) in Uganda. Our study examines short-term fertility desire (wanting a child in the next 12 month) as the primary outcome, as this measure is most likely to achieve consensus within a couple, and to influence behaviors such as contraceptive use. There is also less evidence highlighting how sero-status may interplay with fertility desires in the short term. We examined the influence of male and female sero-positive status on their short-term fertility desire, as well as the influence of other individual and partner characteristics. These additional characteristics include: individual HIV status, partner HIV status, age in years, partner age in years, education attainment, number of living children, community area and socioeconomic status (SES using a series of questions about household possessions such as radios and the use of modern building materials to construct houses).[19] We compared the short-term fertility desires for female and male partners in current relationship to HIV status, both in the general adult sample and then stratified by parity.

## Methods

### Study design

The Rakai Community Cohort Study (RCCS) is an open population-based cohort which enrolls all consenting adult residents aged 15–49 in approximately 50 administrative communities distributed throughout the Rakai District, Uganda.[20] It is nested within the Rakai Health Sciences Program which over the last 30 years has provided continuous access to HIV prevention, care, treatment and support to its members in Rakai, Uganda. In each study data collection round, a household census is first performed in the participating communities describing household information, including births, death, age-in, age-out and migration of the members to identify eligible individuals for the survey. The household census also describes the household possession and dwelling characteristics, which are used for the construction of the socioeconomic status of the household.[21] Among eligible and consenting adults, a detailed survey is conducted to collect comprehensive information on sociodemographic, behavioral, sexual network, mobility, health and service utilization. For individuals who take the survey for the first time, a baseline questionnaire is administered, while for individuals who have already taken the baseline questionnaire, a follow-up questionnaire is given. The questionnaires evolve over study data collection rounds to reflect new research and program innovations of HIV/AIDS prevention and treatment (e.g. availability of treatments, decreasing mortality). Both female and male partners participate in the survey rounds in the same communities on similar dates. After survey completion, the participants will provide a blood sample for HIV testing. Approximately 18,000 individuals participate in each survey round, with a response rate among age eligible persons of about 78%. Among those who complete the surveys, compliance to provision of specimen for HIV testing is over 90%. Prior to all fieldwork, ethics and regulatory oversight for the RCCS was provided by the Uganda Virus Research Institute’s Research Ethics Committee, the Uganda National Council for Science and Technology (UNCST), the Columbia University IRB, and the Western IRB in the United States. The Columbia Unviersity IRB specifically approved this study.

For this paper, we analyzed data collected in the 15^th^ data collection round (2011–2013), from 40 participating communities, due to two major reasons: (1) the questionnaire included more information about different sexual partners (up to four sexual partners), allowing for the linking and of both partner surveys and blood samples, and; (2) more robust reproductive health measures were added into this questionnaire for both women and men. The analyses were restricted to identified couples, defined as being married and/or consensually cohabitating. Of the interviewed sample, approximately ~95% respondents were linked with a single partner (while they might have unrecorded multiple partners). Other individuals linked with multiple partners were considered as separate couples. From 9,251 women and 9,990 men in Round 15, we identified 2,291 couples in which both member of the dyad were enrolled in the RCCS cohort, both had a valid response on short-term fertility desire, and in which the female partner was not pregnant at the time of interview. Over 95% of the couples sample were cohabiting couples.

### Analyses

#### Outcome variable

The main outcome variable was short-term fertility desire. In the male survey (both baseline and follow-up surveys), this variable was obtained by asking “Would you like to have a child in the next 12 months?” In the female baseline survey, the variable was obtained by asking, “Would you like to become pregnant in the next twelve months?” In the female follow-up survey, this variable was not directly asked but was constructed from two separate questions, “Would you like to have (a/another) child?” and, “How long would you like to wait from now before the birth of (a/another) child?” Participants who would like to have a/another child in “less than 1 year” were coded as yes (wanting to have a child in the short-term).

#### Primary predictors

The primary predictor of interest was the HIV sero-status of couples, constructed from both individual and partner data. The four categories were both sero-positive (F+M+), both sero-negative (F-M-), female sero-positive (F+M-) and male sero-positive (F-M+). HIV sero-status was determined based on a validated three assay rapid test algorithm in the field (Determine and Stat Pak run in parallel with Unigold as a tiebreaker).[22,23] All rapid test positives, and discordant or weak positive bands were assessed by two enzyme immunoassays (EIAs: Vironostika and Bio Rad). Polymerase chain reaction (Abbott RealTime HIV-1 PCR) confirmation was conducted on new sero-converters or samples with discordant EIAs. The sero-status results from rapid tests were available right after completing the survey, and the confirmative results were available after a few business days. At that time, participants could choose whether or not to receive their sero-status results, and/or decide whether or not to inform their partners of their sero-status.

#### Other variables

The demographic characteristics that we reported in these analyses were collected through a household census and the RCCS questionnaire. Individual and household characteristics included: individual’s age and partner’s age (15–19 year old, 20–24 year old, 25–34 year old and 35–49 year old), number of living children (0–2, 3 or more), educational attainment (never attended school, attended primary, or post-primary education), and socioeconomic status (SES). For SES, a scale was created (low, middle and high), constructed from number of household possessions and home construction materials,[24] and type of community (rural, trading or fishing). For women, the parity variable was directly asked to women during the survey administration, while for men, the variable was constructed from census data that included household information for men. Census data was used for men for parity since the initial RCCS survey design did not ask men about their existing number of children.

For the analysis, we started by examining the level of agreement in short-term fertility desires between female and male partners by using Cohen's kappa coefficient, both in all couples and stratified by serostatus. Cohen's kappa coefficient measures level of agreement within a couple and is a more robust measure than simple percent agreement calculation, since the kappa takes into account the likelihood that the agreement occurred by chance. Kappa take on a value between 0 and 1, with a higher score signifying greater level of agreement.

Descriptive analyses were performed to examine characteristics of the sample and describe their associations with short-term fertility desires. Poisson regression was used to estimate the rate ratio (RR) of having a short-term fertility desire by individual and partner characteristics (couple’s HIV status, age in years, partner age in years, education attainment, number of living children, community area and SES). Poisson regression was selected since the prevalence of short-term fertility desire was high and the odds ratio (OR) obtained from logistic regression may likely overestimate the relative risk. Unadjusted model, adjusted model with main effects, and adjusted model adding interactive effects of parity and couples’ HIV status were then conducted and presented below. In multiple regression, backwards selection technique was used to select variables with significant impact on short-term fertility desires. All analyses were stratified by gender.

Analyses were conducted using STATA version 14 [College Station, TX: StatCorpLLC].[25]

## Results

We analyzed 2,291 couples; of these, 46.3% were from smaller rural trading villages (rural) with approximately 12% HIV prevalence. Approximately 18.5% were from larger trading centers serving as local and regional transport hubs (trading) with approximately 20% HIV prevalence, while the remaining 35.2% were from high risk fishing communities on Lake Victoria (fishing) with approximately 42% HIV prevalence. Based on household dwelling, 48.8% were from high SES households, 25.1% were from middle SES and 25.8% were from low SES households. About 70.1% couples were both HIV sero-negative, 16.2% were both HIV sero-positive, 7.6% were female positive only, and 6.2% were male positive only. Female partners were generally younger than their male partners: about 8.1%, 23.6%, 44.4% and 20% of women were 15–19 years, 20–24 years, 25–34 years and 35–49 years, respectively, compared to 0.7%, 9.7%, 44.6% and 44.9% of men in these same age categories. Education attainment were comparable among female and male partners in current relationships: 6.7% of women and 5% of men never went to school, while 65.4% of women and 68.4% of men had primary education, and 27.8% of women and 26.6% of men had post-primary education. As mentioned earlier, more men (52.1%), than women (0.9%), had missing information for the number of living children (since men’s data were extracted from census data rather than the RCCS survey). Among those with reported living children, female and male partners reported similar number of children, with a few more men having a larger number of children (> = 3): 50.3% women and 52.7% men had 3 or more children.

Table 1 presents the observed agreement and disagreement levels around short-term fertility desires within couples, presented by total sample and sero-status stratification. Of all 2,291 couples in the sample, the observed disagreement proportion over fertility desires was 29% (Kappa coefficient = 0.37; p-value<0.001). The highest level of disagreement occurred among couples with a female sero-positive partner and a male sero-negative partner, with an observed disagreement proportion of 37% (Kappa coefficient = 0.29; p-value<0.001). In summary, the short-term fertility desire of men and women showed low to moderate agreement in general, with many couples not in consensus about their short-term fertility desires. Moreover, couples with female sero-positive and male sero-negative had the lowest levels of agreement over their short-term fertility desires.

**Table 1:** Dependency of couple's short-term fertility desire by sero-status in non-pregnant couples.

	Short-term fertility desire				% of couples with discordant fertility desire	Kappa coef.	P-value
	F(No) M(No)	F(Yes) M(No)	F(No) M(Yes)	F(Yes) M(Yes)			
Sero-status							
F- M-	939	85	344	237	0.27	0.36	<0.001
F+M-	55	16	48	56	0.37	0.29	<0.001
F- M+	68	7	34	32	0.29	0.40	<0.001
F+ M+	144	27	98	101	0.34	0.34	<0.001
Total	1206	135	524	426	0.29	0.37	<0.001

Table 2 presents descriptive characteristics, unadjusted and adjusted Poisson regression models with main effects, in addition to adjusted Poisson regression models with parity and seropositive status interactive effects (as reported by non-pregnant women). Among the 2,291 non-pregnant women, 24.5% reported they would like to have a child in the next 12 months. At the couple level, 41.1% women in F+M- couples, 34.6% in F+M+ couples and 27.7% in F-M+ couples wanted to have a child in the next 12 months, in comparison to 20.1% women from F-M- couples.

**Table 2:** Short-term fertility desire in non-pregnant women, Rakai, Uganda, 2011–2013.

	N	% *	Possion Regression											
Total	2,291	24.5												
			Unadjusted				Adjusted (Main effect)				Adjusted (Interaction)			
			RR	95% CI		P-value	RR	95% CI		P-value	IRR	95% CI		P-value
**Couple HIV status**														
F- M-	1,605	20.1	ref	ref	ref	ref	ref	ref	ref	ref				
F+ M-	175	41.1	2.05	1.68	2.51	<0.001	1.58	1.30	1.92	<0.001				
F- M+	141	27.7	1.38	1.04	1.83	0.027	1.23	0.93	1.63	0.142				
F+ M+	370	34.6	1.72	1.45	2.05	<0.001	1.33	1.12	1.58	0.001				
**Number of living children**														
0–2	1,074	37.7	ref	ref	ref	ref	ref	ref	ref	ref				
3 or more	1,197	12.9	0.38	0.32	0.44	<0.001	0.35	0.29	0.41	<0.001				
NA	20	10.0	0.19	0.05	0.72	0.014	0.29	0.08	1.06	0.062				
Couple HIV status by Parity (0–2 living children)*														
F- M-	745	30.5	ref	ref	ref	ref					ref	ref	ref	ref
F+ M-	90	58.9	1.93	1.58	2.37	<0.001					1.57	1.28	1.93	<0.001
F- M+	61	41.0	1.35	0.98	1.85	0.070					1.15	0.83	1.59	0.403
F+ M+	178	56.2	1.84	1.56	2.18	<0.001					1.44	1.20	1.72	<0.001
Couple HIV status by Parity (3 or more living children)*														
F- M-	846	11.1	ref	ref	ref	ref					ref	ref	ref	ref
F+ M-	85	22.4	2.01	1.30	3.12	0.002					1.61	1.04	2.51	0.034
F- M+	77	18.2	1.64	0.98	2.73	0.059					1.46	0.88	2.40	0.139
F+ M+	189	14.3	1.29	0.86	1.91	0.216					1.04	0.70	1.55	0.856
**Age in years**														
15–19	185	40.5	1.80	1.47	2.21	<0.001								
20–24	540	29.8	1.33	1.12	1.57	0.001								
25–34	1,107	22.5	ref	ref	ref	ref								
35–49	459	16.6	0.74	0.58	0.93	0.010								
**Partners age in years**														
15–19	17	47.1	1.69	1.01	2.82	0.046								
20–24	223	37.7	1.35	1.11	1.64	0.003								
25–34	1,022	27.9	ref	ref	ref	ref								
35–49	1,029	17.9	0.64	0.54	0.76	<0.001								
**Education Attainment**														
Never go to school	154	29.9	1.17	0.91	1.52	0.226	1.13	0.88	1.44	0.337	1.13	0.89	1.44	0.330
Primary education	1,499	25.5	ref	ref	ref	ref	ref	ref	ref	ref	ref	ref	ref	ref
Post primary education	638	20.9	0.82	0.69	0.97	0.024	0.84	0.71	0.99	0.038	0.84	0.71	0.99	0.037
**Area**														
Rural	1,061	17.4	ref	ref	ref	ref	ref	ref	ref	ref	ref	ref	ref	ref
Trading	423	18.4	1.06	0.83	1.34	0.647	1.02	0.81	1.29	0.858	1.03	0.81	1.29	0.829
Fishing	807	36.9	2.12	1.81	2.48	<0.001	1.73	1.47	2.04	<0.001	1.74	1.47	2.04	<0.001
**SES**														
High	1,119	23.6	ref	ref	ref	ref								
Middle	576	18.9	0.80	0.66	0.98	0.030								
Low	590	31.9	1.35	1.15	1.58	<0.001								

* % of having short-term fertlity desire

In the unadjusted Poisson models, both women’s HIV status and their male partners’ HIV status were significantly associated with having a short-term fertility desire (unadjRR = 2.05, p-value<0.001 for F+M-; unadjRR = 1.38, p-value = 0.027 for F-M+; unadjRR = 1.72, p-value<0.001 for F+M+; in comparison with F-M-). In the adjusted main effects model which controls for all significant demographic variables (age, educational attainment, number of living children and area of residence), only women’s own HIV status influenced their short-term fertility desire while their male partners’ HIV status did not (adjRR = 1.58, p<0.001 for F+M-; adjRR = 1.33, p = 0.001 for F+M+; in comparison with F-M-). The strongest covariate was the number of living children, with 3 and more children having an adjRR of 0.35 (95%CI 0.05–0.14), in comparison to women with only 0–2 living children.

Accounting for the interactive effect of parity and serostatus, their main effects were left out of the model during variable selection. The same pattern as with the overall sample was observed when the number of living children was small (0–2 children; [adjRR = 1.57, p<0.001 for F+M-; adjRR = 1.44, p<0.001 for F+M+; in comparison with F-M-]). The difference in short-term fertility desires between F+M+ and F-M- couples disappeared for women whose number of living children was large (3 or children) (adjRR = 1.04, p = 0.856). Having post-primary education (adjRR = 0.84, p = 0.038) and living in fishing villages (adjRR = 1.73, p<0.001) were also significant predictors in the adjusted main effects model, and their effect remained consistent in the interaction model. The impact of age–of both women and their partners—disappeared in adjusted models, after controlling for number of living children and serostatus.

Table 3 presents the same set of analyses using data reported by men. The sample size of male partners was the same as female partners (n = 2,291), with 41.5% of men reporting the desire to have a child in the next 12 months, which was much higher than women’s short-term fertility desires (24.5%). Stratified by couple’s HIV status, 59.4% men from F+M- couples, 53.8% from F+M+ couples, and 46.8% from F-M+ couples wanted to have a child in the next 12 months in comparison to 36.2% men from F-M- couples. These data suggest a similar pattern across gender in short-term fertility desire by HIV status: both genders had the highest short-term fertility desire when the female partner was HIV positive (F+M- couples) and the lowest when both partners were HIV negative (F-M- couples). In the unadjusted and adjusted main effects models, both a man’s personal HIV status and his female partner’s HIV status were significantly associated with having a short-term fertility desire. When interactive effects with parity was considered, the impact of sero-positive status remained similar when the number of living children was small (0–2 children), but disappeared when the number of living children was 3 or more (all four serostatus groups were not significantly different from each other). Having post-primary education had a negative association with short-term fertility desires in both adjusted models. These male partner results were consistent with data from female partners, earlier presented. The age effect was fully controlled for in the adjusted interactive model, but had a remaining effect in the adjusted main effects model, as younger men more likely to want another child in the next twelve months.

**Table 3:** Short-term fertility desire in men, Rakai, Uganda, 2011–2013.

	N	%*	Possion Regression											
Total	2,291	41.5												
			Unadjusted				Adjusted (Main effect)				Adjusted (Interaction)			
			IRR	95% CI		P-value	IRR	95% CI		P-value	IRR	95% CI		P-value
**Couple HIV status**														
F- M-	1,605	36.2	ref	ref	ref	ref	ref	ref	ref	ref				
F- M+	141	46.8	1.29	1.07	1.56	0.007	1.23	1.03	1.46	0.022				
F+ M-	175	59.4	1.64	1.43	1.89	<0.001	1.42	1.24	1.63	<0.001				
F+ M+	370	53.8	1.49	1.32	1.67	<0.001	1.26	1.13	1.41	<0.001				
**Number of living children**														
0–2	545	67.2	ref	ref	ref	ref	ref	ref	ref	ref				
3 or more	552	38.2	0.57	0.50	0.64	<0.001	0.65	0.57	0.74	<0.001				
NA	1,194	31.2	0.47	0.42	0.52	<0.001	0.56	0.50	0.63	<0.001				
**Couple HIV status by Parity (0–2 living children)**														
F- M-	303	30.5	ref	ref	ref	ref					ref	ref	ref	ref
F- M+	35	58.9	1.45	1.21	1.74	<0.001					1.40	1.17	1.68	<0.001
F+ M-	64	41.0	1.45	1.25	1.68	<0.001					1.41	1.22	1.64	<0.001
F+ M+	143	56.2	1.36	1.19	1.55	<0.001					1.29	1.13	1.48	<0.001
**Couple HIV status by Parity (3 or more living children)**														
F- M-	354	11.1	ref	ref	ref	ref					ref	ref	ref	ref
F- M+	41	22.4	1.08	0.72	1.62	0.71					1.06	0.70	1.61	0.767
F+ M-	53	18.2	1.20	0.86	1.68	0.29					1.18	0.84	1.67	0.329
F+ M+	104	14.3	1.17	0.90	1.52	0.24					1.13	0.87	1.47	0.349
**Age in years**														
15–19	17	76.5	1.66	1.27	2.18	0.000	1.36	1.01	1.82	0.044				
20–24	223	57.0	1.24	1.09	1.41	0.002	1.09	0.96	1.24	0.198				
25–34	1,022	46.0	ref	ref	ref	ref	ref	ref	ref	ref				
35–49	1,029	33.0	0.72	0.64	0.80	0.000	0.83	0.74	0.93	0.001				
**Partners age in years**														
15–19	185	56.8	1.39	1.21	1.61	<0.001								
20–24	540	45.0	1.10	0.98	1.24	0.096								
25–34	1,107	40.7	ref	ref	ref	ref								
35–49	459	32.9	0.81	0.70	0.94	0.005								
**Education Attainment**														
Never go to school	114	57.0	1.32	1.12	1.57	0.001	1.16	0.99	1.37	0.067	1.09	0.92	1.28	0.311
Primary education	1,567	43.1	ref	ref	ref	ref	ref	ref	ref	ref	ref	ref	ref	ref
Post primary education	610	34.3	0.79	0.70	0.90	<0.001	0.88	0.78	0.99	0.035	0.78	0.66	0.93	0.005
**Area**														
Rural	1,061	34.1	ref	ref	ref	ref								
Trading	423	38.1	1.12	0.96	1.29	0.146								
Fishing	807	52.9	1.55	1.39	1.72	0.000								
**SES**														
High	1,126	37.6	ref	ref	ref	ref								
Middle	573	38.9	1.04	0.91	1.18	0.586								
Low	590	51.4	1.37	1.23	1.52	0.000								

* % of having short-term fertlity desire

## Discussion

In these analyses, we found that men and women in current relationships influenced one another’s short-term fertility desires, however their agreement on what these desires should be was moderate. The lowest agreement was among couples with female sero-positive and male sero-negative partners. This group had the highest proportion of both types of disagreement (women’s desires vs. men non-desires, and men’s desires vs. women non-desires), compared to all couple’s sero-status categories. The lack of consensus in couple’s short-term fertility desires may be a lack of spousal communication around fertility desires and sexual and reproductive health, in addition to a reflection of potential instability in their relationship (perhaps as a result of HIV status). When deciding on potential pregnancies, lack of couple communication–coupled with the presence of potential relationship conflict—may also be complicated by use of family planning methods to reduce HIV transmission between partners and children.

These analyses also demonstrated that HIV status did impact short-term fertility desires–and also that gender and parity influence this relationship. When the number of living children was low, a man’s positive sero-status only had a significant association with his increased fertility desire. However, a woman’s positive sero-status appeared to have a much stronger impact: women’s positive sero-status was both associated to her increased fertility desire, in addition to her male partner’s. Yet when the number of living children was high, a man’s sero-status became irrelevant while a woman’s positive sero-status still had a significant positive effect on her own fertility desire.

Despite the growing body of literature on HIV and fertility, no consensus has been reached on how gender influences the interplay between fertility desires and HIV status,[26–28] and it is also unclear what are the underlying reasons for observing increased short-term fertility desires given sero-positive status in Rakai. An in-depth interview of discordant couples in Kenya also found that, “while the goal for childbearing was unchanged, conception became an urgent desire so that both partners could experience childrearing together while the HIV-infected partner was still healthy.”[4] Previous studies among women in Rakai have found that HIV positive status was linked with decreased lifetime total fertility desires.[29] This study found HIV positive status is associated with increased fertility desire in the short term (next 12 month). The nuance to this finding is that seropositive women would like to have less children in total, however they desire but more children in the short-term when compared to their seronegative counterparters, this perhaps suggests a desire to speed up childbearing to reach their desired total fertility rate, in the face of potentially deteriorating health due to HIV/AIDS. Little information is available for men to understand their motivations behind short-term and long-term fertility deisres. Future family planning policy and programs should consider the potential influence of partner HIV status in fertility-related decisions and potential family planning use. For example, women with discordant fertility desires with their partners have been found to seek covert contraceptive use due to fear of being ostracized by their communities.[30] Family planning programs should be aware of such dynamics to reduce stigma and discrimination as a result of concurrent HIV infection and discordant fertility desires.

### Limitations

A limitation of this paper is that the question on short-term fertility desire for both members of a couple was only available in one round of data and therefore, could not be examined longitudinally. While the covariate effects on male and female short-term fertility desires were evaluated, the process in which these covariates influence the agreement and disagreement of the couples was not clear. We also did not link short term fertility desire with contraceptive use and subsequent pregnancies but this could be a potential extension of this work. A lack of data and information on previous pregnancies (such as the distance from last birth and any child with this partner) is also a limitation to this study. Another key variable for this study was that the parity variable was constructed, not directly asked, for men, generating a big proportion of missing values. This study did not include fertility-related data among non-cohabiting couples. Finally, this study uses data nested in the Rakai Health Sciences Program and its findings may not be fully generalizable to other HIV patients in Uganda or elsewhere in SSA that have limited access to HIV programs.

### Implications

Programs that sit at the intersection between HIV and family planning have often failed to consider the dyadic nature of reproduction or the role of male intentions in fertility. In the cultural and economic context of SSA, men tend to be the dominant decision-maker in household and family activities, including issues relating to fertility and family planning. Thus, it is essential to understand fertility desires at a couple level–and across female and male partners—within contexts where HIV prevalence is high. Our study added to the existing literature on this topic and collected information from both partners within the context of a generalized HIV epidemic, to account and control for men and women’s HIV sero-status and short-term fertility desires.

We found that men and women’s short-term fertility desires were weakly to moderately correlated within a couple context, and both women and men’s short-term fertility desires were more strongly associated with women’s HIV status, rather than men’s HIV status. Policy and programs should consider these realities in fertility-related decisions to more effectively meet couples’ needs in sexual and reproductive health.
